# Sophorolipids production from rice straw via SO_3_ micro-thermal explosion by *Wickerhamiella domercqiae* var. *sophorolipid* CGMCC 1576

**DOI:** 10.1186/s13568-016-0227-7

**Published:** 2016-08-27

**Authors:** Xin-ge Liu, Xiao-jing Ma, Ri-sheng Yao, Chun-yu Pan, Hua-bing He

**Affiliations:** 1 School of Biological and Medical Engineering, Hefei University of Technology, 193 Tunxi Road, Hefei, 230009 Anhui China; 2 Anhui BBCA Chemical Equipment Co. LTD, Bengbu, 233010 China

**Keywords:** Sophorolipid, RSHH, Lac SL/tot SL, Broth pH, Correlation

## Abstract

A novel lignocellulose material, holocellulose from rice straw via the pretreatment of SO_3_ micro-thermal explosion, was developed to produce sophorolipids (SLs) with *Wickerhamiella domercqiae* var. *sophorolipid* CGMCC 1576. The influence factors of inoculum dose, yeast extract concentration and pH regulators (chemical regents used for adjusting/influencing pH) was investigated and discussed. Results showed that *W. domercqiae* can grow in the rice straw holocellulose hydrolysate, and acquire relative high SL yield of 53.70 ± 2.61 g/L in shake flask culture. Inoculum dose, yeast extract concentration and pH regulator made obvious influence on fermentation parameters, especially on final broth pH and SLs production. Furthermore, there is a strong negative linear correlation existing between final broth pH and lactonic SL or ratio of lac SL/tot SL. Additionally, comparison between SL production and non-glucose carbon sources, culture methods, microbes in previous reports was carried out. These results will be benefit for acquiring SL mixture with suitable lac SL/tot SL ratio for specific purpose and scope economically.

## Introduction

Sophorolipids (SLs), an extracellular biosurfactant, are being applied in fields of detergent (Cox et al. 2013; Lee et al. 2014), petroleum (Sirola 2010), cosmetic (Hillion et al. 1998; Morya et al. 2013), pharmaceutical (Chen et al. 2006; Morya et al. 2013; Singh et al. 2016), food processing (Cooper and Paddock 1984; Liu et al. 2009), environment industries (Sirola 2010), nano material (Pandey et al. 2016) and fermentation engineering (Gross et al. 2015) for their potential characteristics. They have comparable surface activity, biodegradability, biocompatibility, and low toxicity compared with chemical surfactants (Banat et al. 2000; Lee et al. 2008). Nonpathogenic yeasts of *Candida apicola*, *Rhodotorula bogoriensis*, *Torulopsis gropengiesseri*, *Candida bombicola*, *Wickerhamiella domercqiae*, *Torulopsis bombicola*, *Pichia anomala*, *Candida batistae*, *Candida rugosa*, *Candida stellata*, *Candida floricola* etc. were reported to produce SLs (Kurtzman et al. 2010; Imura et al. 2010; Chandran and Das 2011; Bogaert and Soetaert 2011).

Ordinary, SLs occur as a mixture of lactonic and acidic SL molecules, lactonic SLs show better surface tension reducing propriety and biological activities, while the acidic ones display better foam formation ability and solubility and allow further modification at the free carboxylic acid end (Concaix 2003; Chen et al. 2006; Hu and Ju 2001; Maingault 1999). Although SLs was first published in 1961 (Gorin et al. 1961), they gained increased attention as growing environmental awareness in the last two decades. Currently, both choosing new substrates to lower production cost of SLs and selectively synthesis of SLs by optimizing fermentation conditions are hot topics.

SLs-producing yeasts can grow on variety of saccharic and lipidic feed-stocks to accumulate SL mixture. Glucose and oleic acid are common used substrates. One of major study on SLs is to explore new resource to replace glucose. Non-lignocellulose material, such as whey (Otto et al. 1999), soy molasses (Solaiman et al. 2004, 2007), honey (Pekin et al. 2005), cassava starch (Thaniyavarn et al. 2008), sugarcane molasses (Daverey and Pakshirajan 2009; Takahashi et al. 2011), sweet water (Wadekar et al. 2012), glycerol (Bhangale et al. 2014) had been studied previously. However, SL yields were mostly lower when different sugars or low-cost substrates were employed, and the cost-reducing effects were inconspicuous compared to SLs obtained from glucose.

Lignocellulosic material is one of the most abundant renewable resources. Hydrolysate of delignified corncob residue (DCCR) was firstly introduced for SLs production by Ma et al. (2013). Total SL yield of 32.79 g/L and 36.25 g/L was obtained from DCCR without/with detoxification, respectively. This report expanded the range of raw materials for SLs production. Furthermore, to eliminate the wastewater appended in DCCR, Masaaki Konishi et al. (2015) developed SLs from corncob hydrolysate (CCR), with a SL yield of 49.20 g/L. They also found that excess sulfuric acid used during corncob pretreatment would increase the content of furfural in hydrolysate. Additionally, Samad et al. (2014) obtained 84.60 g/L of SLs from bagasse by continuous fermentation with a fermentation time of 240 h. Inhibition effects of lignin on SLs production were also verified. Hence, lignocellulosic materials are potential resources for SL production and pretreatment was essential for production improving.

Although corncob is easier to be utilized than other raw materials, rice straw and wheat straw are the most abundant biomass resources to be exploited and used in the world (Binod et al. 2010). In order to make cellulose more accessible, pretreatment of straw was used to break the lignin seal and disrupt the crystalline structure of cellulose. The pretreatment method of “SO_3_ micro-thermal explosion” was proposed by our group and could be applied to pretreat rice straw, wheat straw and other lignocellulose. Preliminary work has proved that this method has advantages of high lignin removal rate, low consumption, less inhibitors and little wastewater generating (Yao et al. 2011; Li et al. 2012).

For research influence factors on SLs production and lac SL/tot SL ratio in SL mixture, some studies regarding carbon sources, nitrogen sources, pH, cultivation methods and strains, etc. were available (Cooper and Paddock 1984; Zhou et al. 1992; Casas and García-Ochoa 1999; Göbbert et al. 1984; Stüwer et al. 1987). Our previous work also indicated that inorganic nitrogen sources could significantly inhibit lactonic SL production and addition of pH regulators could enhance lactonic SL production by increasing broth pH value for *W. domercqiae*. However, further research is needed to more accurately assess the potential beneficial and harmful effects of nitrogen source and pH regulator on SLs production.

The aim of the present work was to explore inexpensive substrate for SL production and obtain SL mixture with appropriate lac SL/tot SL ratio for various use. Firstly, rice straw pretreated by the method of SO_3_ micro-thermal explosion was introduced for SLs production. Then, the fermentation process and the influence factors on SLs accumulation by *W. domercqiae* in rice straw holocellulose hydrolysate (RSHH) were investigated and discussed. Finally, the relationship between the final broth pH and lactonic SL or lac SL/tot SL ratio was fitted. The current study is an attempt to address substrates and main parameters of SL fermentation to arrive at an economically SL-producing procedure.

## Materials and methods

### Rice straw pretreatment and enzymatic hydrolysis

Rice straw was obtained from a local farm in the suburb of Hefei, China, which was harvested in 2015 and pretreated according to our previous study. Firstly, rice straw was cut into small pieces of about 2–3 cm in length. Then holocellulose was obtained with the pretreatment of SO_3_ micro-thermal explosion, ammonia wash, alkaline wash, water wash and drying processes, successively (Yao et al. 2011).

For enzymatic hydrolysis experiments, crude KDN cellulase solution (KDN Biotech Co., Ltd., Qingdao, China) with filter paper activity (FPA) of 67 FPU/mL was used. Before enzymatic hydrolysis, rice straw holocellulose (RSH) was desiccated at room temperature and subsequently milled to power in preparation. RSHH was obtained by hydrolyzing 10 % of RSH with the enzyme dose of 25 FPU/g of dry RSH in 0.2 M sodium acetate buffer (pH 4.8) at 45 °C and 150 rpm for 72 h. After hydrolysis, the liquid fraction was collected by centrifugation and the cellulase was inactivated at 80 °C for 0.5 h. All RSHH was stored at 4 °C prior to use.

### Microorganism and growth condition

*Wickerhamiella domercqiae* var. *Sophorolipid* CGMCC 1576 was offered by Professor Song of Shandong University and now preserved in China General Microbiological Culture Collection Center (CGMCC). The seed inoculum was prepared by growing the organism in seed medium on a rotary shaker at 220 rpm for 16 h at 30 °C. The seed medium contained glucose 20, peptone 20 and yeast extract 10 (g/L). Then the seed culture was transferred to different fermentation media (50 mL in 300 mL flask) and cultivated for 7 days at 220 rpm at 30 °C.

The chemical defined fermentation medium, which containing ingredients (w/v, g/L) of glucose 60.00, yeast extract 3.00, KH_2_PO_4_ 1.00, Na_2_HPO_4_·12H_2_O 1.00, MgSO_4_·7H_2_O 0.05, and oleic acid 60.00 (v/v, mL/L), was used for SLs production and set as the control group. For hydrolysate fermentation medium, rice straw holocellulose hydrolysate (RSHH) which containing 60.00 g/L of glucose was used to replace glucose as carbon source. Then, effects of different seed inoculum dose, yeast extract concentration and pH regulator on SLs production and lac SL/tot SL ratio in RSHH fermentation medium was investigated. The experiment was carried out in triplicate, respectively. Different seed inoculum dose of 1, 2, 4 %, yeast extract in different concentration (g/L, 0.00, 0.05, 0.10, 0.15, 0.20, 0.25, 0.30) and different pH regulator (NaOH, K_2_CO_3_, CaO, AlCl_3_, C_6_H_8_O_7_, Na_3_C_6_H_5_O_7_·2H_2_O) were added at the beginning of the cultivation. The other ingredients in RSHH medium were the same as that in the control group.

### Analysis of chemical and elementary composition

The chemical composition of rice straw and obtained holocellulose was analyzed by the method of NREL. The elementary analysis was determined by elemental analyzer (Elementar, Germany). The experiments of chemical composition and elementary analysis all were carried out in triplicate and the results reported are mean ± SD of the three independent experimental results.

### Analysis of scanning electron microscope images

Scanning electron microscope (SEM, Hitachi, Japan) instrument was used to observe morphology variation of rice straw and confirm the pretreatment effects of SO_3_ micro-thermal explosion. The images were collected under the magnification of 1000 and 200 times, at the accelerating voltage of 20 kV.

### Determination of residual glucose, pH, biomass, SL production

Glucose in hydrolysate and residual glucose in broth was measured by SBA-40C bio-sensor analyzer (Shandong Academy of Sciences, Shandong, China) with an injection volume of 25 μL. Broth pH value was measured by PHS-3C pH meter (Shanghai Shengci Instrument co., LTD, China). For biomass determination, 1.0 mL of culture broth was mixed with two volumes of *n*-butanol/ethanol/chloroform (10:10:1), and centrifuged at 8000 rpm for 10 min. The solid residue was washed twice with distilled water, and dried at 50 °C to a constant weight. All data were presented as the mean of three readings. For lactonic SL determination, two volumes of ethyl acetate were added to 0.5 mL fermentation broth firstly, after shaking and extracting, the lactonic SL in organic phase was measured by anthrone method. For total SL determination, 1.0 mL of ethanol was added to 0.5 mL broth to dissolve SLs, then the solution was centrifuged at 8000 rpm for 5 min, and total sugar content in the supernatant was quantified by anthrone method. Total SL production was calculated according to the glucose standard curve with glucose content of total sugar content minus residual glucose content (Ma et al. 2011). Lac SL/tot SL was calculated as a ratio of lactonic SL production on total SL production and stated as a percentage (%). The measurements were carried out in triplicate for each fermentation broth and all results reported are the mean of three independent experimental results.

## Results

In the present study, the composition or structural characteristics of holocellulose have been detected and analyzed firstly. And major fermentation influencing factors of SLs production by *W. domercqiae* was studied. Several parameters including substrates, inoculum dose, yeast extract concentration and pH regulator was optimized to attempt to reduce the cost and make it more economical.

### Chemical, elemental and SEM analysis of rice straw and holocellulose

**Fig. 1:**
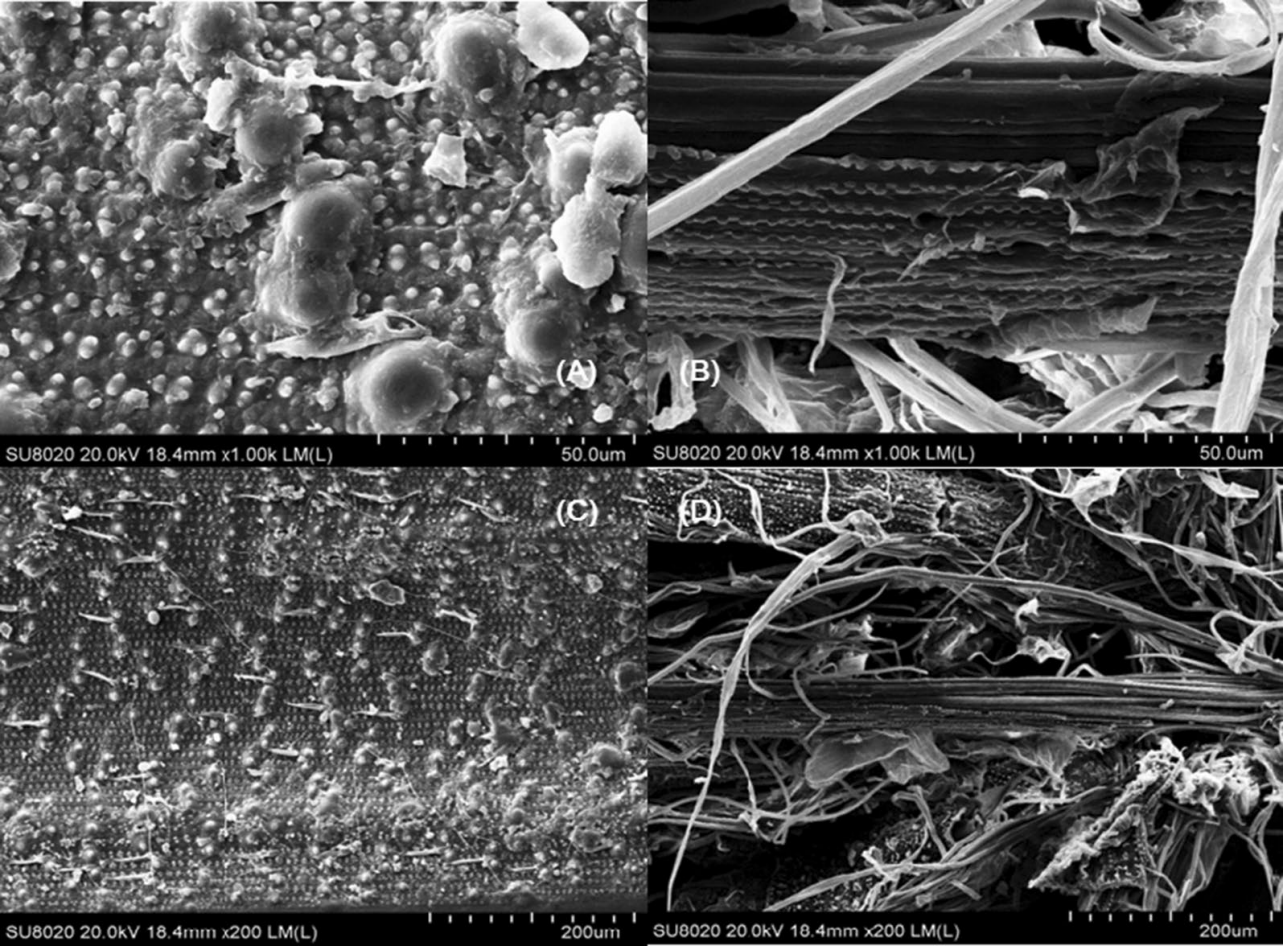
SEM images comparison of rice straw before and after pretreatment. **A** Rice straw ×200; **B** holocellulose ×200; **C** rice straw ×1000; **D** holocellulose ×1000

The differences in chemical, elemental and SEM analysis of rice straw before/after pretreatment were compared and the results were shown in Tables 1, 2 and Fig. 1.

**Table 1:** Chemical composition analysis of rice straw and holocellulose after pretreatment

	Water (%)	Holocellulose (%)		Lignin (%)	Ash (%)
		Cellulose	Hemicellulose		
Rice straw	11.19 ± 0.74	30.46 ± 0.62	25.56 ± 0.83	14.00 ± 0.75	9.76 ± 0.44
Holocellulose	6.60 ± 0.55	59.78 ± 0.77	26.39 ± 0.99	5.77 ± 0.68	1.38 ± 0.57

**Table 2:** Elemental composition analysis of rice straw and holocellulose after pretreatment

	C (%)	H (%)	N (%)	O (%)	S (%)
Rice straw	37.80	5.27	1.18	43.78	0.11
Holocellulose	41.18	6.13	0.83	50.04	0.06

We could see that rice straw contained 56.02 % of holocellulose and 14.00 % of lignin and lignin content reduced to 5.77 % in holocellulose after pretreatment. In other words, the delaminating rate of lignin reached as high as 58.79 % with the pretreatment of SO_3_ micro-thermal explosion. As shown in Table 1, the change of hemicellulose content of rice straw before and after pretreatment was minor, what is mean xylose content could be equal after hydrolyzing. Besides, the content of water and ash was reduced to 6.60 and 1.38 %, respectively. This phenomenon was because that the small molecule or soluble substances were vaporized and washed. The holocellulose content increased to 86.17 %, which is predominantly attributed to the decrease of lignin, water and ash. This outcome would be conductive to enzymatic hydrolyzing by cellulase to obtain more available sugars.

Normally, chemical structure of lignocellulose mainly is carbon skeleton and main elements are hydrogen, oxygen, nitrogen and sulfur (Witczak et al. 2015). The decrease of nitrogen and sulfur will decrease the amount of acid rain formation, which is an environmental hazard. C content increased from 37.80 to 41.18 %, was due to the increase of holocellulose percentage, the same to the O and H content.

In addition, SEM was carried out to investigate the capacity of lignin removing by pretreatment of SO_3_ micro-thermal explosion directly. The microscopic morphology of rice straw before and after pretreatment with different zoom factors (200× and 1000×). Regarding the rice straw (Fig. 1A, C), a compact structure was exposed. As is seen in Fig. 1, the surface of rice straw was covered orderly by different sizes of circular protuberances, which is called lignin, and fibers in rice straw were arranged intensively (Fig. 1A, B). On the contrary, flaking traces and some holes could be observed on the holocellulose surface (Fig. 1B, D). After pretreatment, few lignin protuberances were remained, and compact microstructure was damaged into disorganized (Fig. 1C, D), with the improvement of loose degree of spatial structure. Hence, the efficiency of enzymatic hydrolysis could be increased significantly after the pretreatment.

In summary, the pretreatment we adopted will enhance hydrolysis and saccharification process by partly breaking rice straw structure. In the current work, glucose content in hydrolysate was increased from 15 to 75 g/L for rice straw before and after pretreatment. It was found that the saccharification rate was increased to 400 % in holocellulose compared to rice straw. These test results were in agreement with above analysis. Based on the above mentioned results, experiments were carried out to explore the possibility of SLs production from RSHH without detoxification. The pre-experiments results showed that *W. domercqiae* could utilize RSHH to produce SLs without any pretreatment in a relatively lower yield than the control group. Therefore, further optimization was necessary to obtained higher production and lower cost.

### Effects of inoculum dose on SL production in RSHH

Effects of the inoculum dose on SL production were shown in Table 3, glucose in RSHH could be applied directly for SL production by *W. domercqiae.*

**Table 3:** Effects of inoculum dose on final broth pH, cell growth and SL production

Inoculum dose	Final pH	Residual glu (g/L)	Biomass (g/L)	Total SL (g/L)	Lactonic SL (g/L)	Lac SL/tot SL (%)
Control group	2.55	0.81 ± 0.08	6.53 ± 0.14	40.74 ± 1.60	18.83 ± 0.01	46.22
1 %	5.21	0.63 ± 0.42	5.57 ± 0.09	29.08 ± 3.48	5.28 ± 3.27	18.17
2 %	4.49	0.78 ± 0.21	6.73 ± 0.25	42.33 ± 1.65	8.76 ± 0.46	20.70
4 %	4.26	0.27 ± 0.04	8.53 ± 0.33	37.56 ± 0.27	14.44 ± 0.97	38.46

It was found that, with the increase of inoculum dose, the final broth pH was decreased and the biomass was increased. Sugars (mainly glucose) in RSHH was effectively utilized in the early exponential phase and almost used up at the end of the fermentation.

The optimum inoculum dose in RSHH medium dose for total and lactonic SL production was 2 and 4 %, respectively. Lactonic SL production and proportion of lactonic SL to total SL increased with increasing inoculum dose, which demonstrated that high inoculum dose is more suitable for lactonic SL production in RSHH medium. The highest total SL production of 42.33 ± 1.65 g/L, 3.90 % higher than the control group, was obtained with the inoculum dose of 2 %. Therefore, the optimal inoculum dose was set as 2 % in the follow-up experiments.

### Effects of yeast extract content on SL production in RSHH

Considering the effects of varying YE levels, the growth of yeasts, pH value of final broth and production of SL in RSHH fermentation medium containing different yeast extract (YE) contents were shown in Table 4.

**Table 4:** Effects of yeast extract concentration on final broth pH, cell growth and SL production

YE content (w/w, %)	Final pH	Residual glu (g/L)	Biomass (g/L)	Total SL (g/L)	Lactonic SL (g/L)	Lac SL/tot SL (%)
Control group	2.55	0.81 ± 0.08	6.53 ± 0.14	40.74 ± 1.60	18.83 ± 0.01	46.22
0.00	4.35	1.77 ± 0.01	3.07 ± 0.78	41.53 ± 3.62	10.03 ± 0.84	24.15
0.05	4.03	0.24 ± 0.04	3.55 ± 0.02	49.66 ± 0.99	22.32 ± 2.37	44.94
0.10	4.16	0.14 ± 0.11	4.47 ± 0.28	51.42 ± 0.92	21.60 ± 0.43	42.01
0.15	4.40	0.03 ± 0.00	4.65 ± 0.35	53.70 ± 2.61	22.06 ± 5.33	41.08
0.20	4.46	0.08 ± 0.06	5.64 ± 0.13	50.09 ± 5.92	20.57 ± 1.77	41.06
0.25	4.66	0.18 ± 0.04	6.88 ± 0.46	46.05 ± 4.30	20.12 ± 1.34	43.70
0.30	4.77	0.18 ± 0.00	7.32 ± 0.46	45.83 ± 2.00	18.12 ± 0.48	39.53

As shown in Table 4, glucose in RSHH was almost exhausted in all cases. Yeast growth increased with the increase of yeast extract content, in accordance with the conclusion that sufficient nitrogen source are beneficial for microbe growth. *Wickerhamiella domercqiae* could survive and utilize sugars in RSHH even no extra nitrogen source adding (YE 0.00 %), which might due to the residual cellulase used in hydrolysis process was applied as nitrogen source.

Total SL production increased with the increasing yeast extract concentration under 0.15 % and decreased with further increasing up to 0.30 %. The highest total SL yield of 53.70 ± 2.61 g/L and superior lactonic SL yield of 22.06 ± 5.33 g/L (the highest was 22.32 ± 2.37 g/L) was obtained with the YE addition of 0.15 %, respectively. Furthermore, yield of total SL still reached 41.53 ± 3.62 g/L without YE supplement, 1.94 % higher than that of control group. However, more acidic SL was synthesized and low ratio was obtained in this condition.

All of the final broth pH of RSHH cultures was higher than that of control group and final broth pH value increased with the YE adding content. Except for the one without YE supplement (YE 0.00 %), maximum final broth pH, minimum total SL, lactonic SL and Lac SL/tot SL ratio of 4.77, 45.83 ± 2.00, 18.12 ± 0.48 g/L, and 39.53 % were obtained with 0.30 % of YE addition, respectively. Interestingly, the results also revealed that there could be certain relation between final broth pH, SL production and ratio within limits.

### Effects of pH regulator on SL production in RSHH

In order to verify above speculation that final broth pH is negative related to lactonic SL and Lac SL/tot SL ratio, experiments on effects of different pH regulator on SLs production in RSHH were developed. Different pH regulators divided into acidifying agent, alkaline and buffer salt with the same addition was selected and applied to the RSHH medium, respectively. The influence of different pH regulator on SLs production was shown in Table 5.

**Table 5:** Effects of pH regulators on final broth pH, cell growth and SL production

pH regulator	Final pH	Residual glu (g/L)	Biomass (g/L)	Total SL (g/L)	Lactonic SL (g/L)	Lac SL/tot SL (%)
Control group	2.55	0.81 ± 0.08	6.53 ± 0.14	40.74 ± 1.60	18.83 ± 0.01	46.22
NaOH	4.56	0.81 ± 0.01	9.65 ± 0.01	38.89 ± 1.09	14.91 ± 0.52	38.33
K_2_CO_3_	5.62	2.40 ± 0.14	5.72 ± 0.39	38.27 ± 4.03	8.14 ± 3.46	21.27
CaO	5.71	0.29 ± 0.02	9.92 ± 1.01	40.69 ± 0.88	11.96 ± 4.46	29.38
AlCl_3_	3.89	0.20 ± 0.02	7.05 ± 0.12	50.09 ± 3.64	23.58 ± 4.79	47.08
C_6_H_8_O_7_	4.18	0.17 ± 0.02	9.21 ± 0.08	41.01 ± 0.48	20.03 ± 2.56	48.84
Na_3_C_6_H_5_O_7_·2H_2_O	4.48	0.24 ± 0.00	5.87 ± 0.08	39.52 ± 1.96	16.13 ± 0.79	40.81

The results showed that different pH regulator have different effects on final broth pH and SL production. In general, comparable or higher SL production compared to the control group was acquired with different pH regulator. When the content of pH regulator was kept constant, Lewis acid like AlCl_3_ and mild acid like C_6_H_8_O_7_ were more suitable for lactonic SL production than alkali like NaOH and Lewis base (K_2_CO_3_, CaO, Na_3_C_6_H_5_O_7_·2H_2_O). Meanwhile, high Lac SL/tot SL ratio of 47.08 and 48.84 % was gained, respectively. It is worth noting that the final broth pH dropped significantly to 3.89 than the others when AlCl_3_ was added and lactonic and total SL production reached 23.58 ± 4.79 and 50.09 ± 3.64 g/L, with an increase of 25.2 % and 23.0 % compared to the control group, respectively. Besides, lactonic SL production and Lac SL/tot SL ratio of the medium contained K_2_CO_3_ and CaO was much lower, in accordance with the trend of final broth pH. The results demonstrated that different pH regulator regulates SL production by changing pH value of the culture again.

### Correlation coefficient analysis between final broth pH, lactonic SL and Lac SL/tot SL ratio in RSHH

**Fig. 2:**
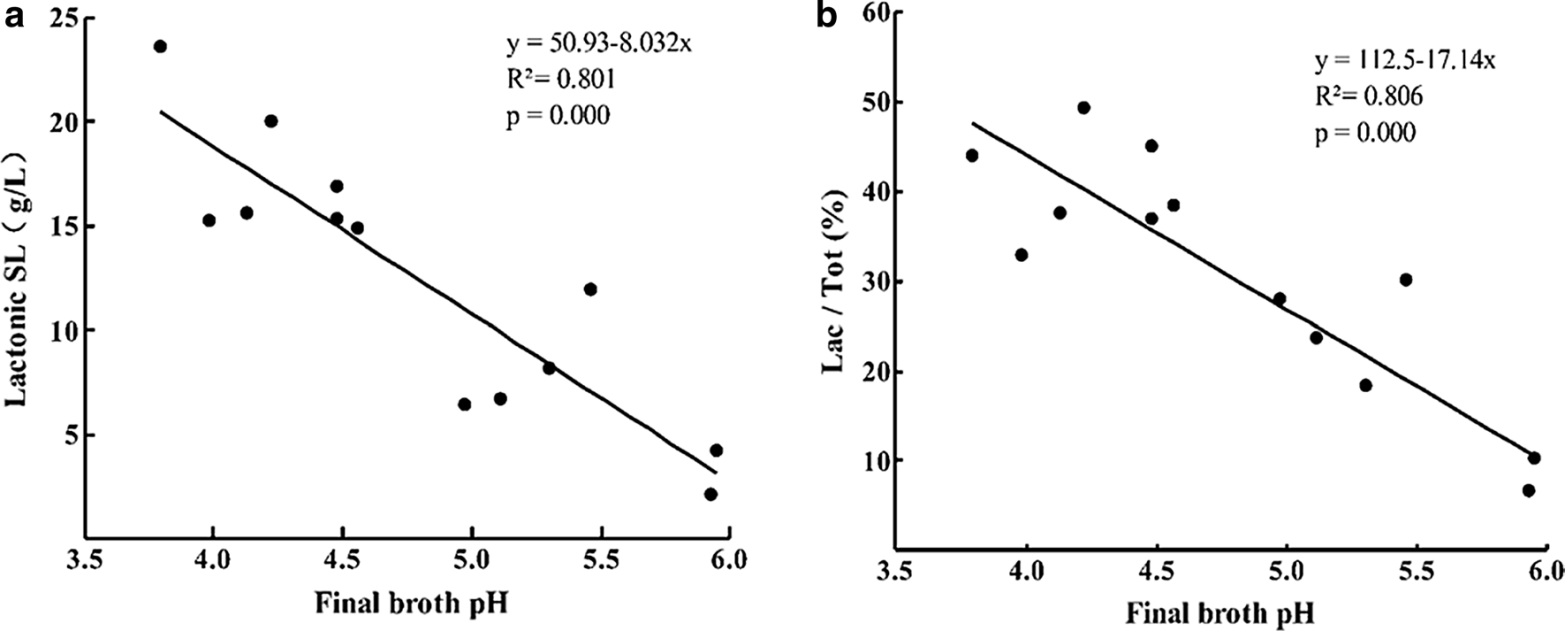
Correlation fitting curve between final broth pH and lactonic SL (**a**) or Lac SL/tot SL ratio (**b**)

All the results above-mentioned suggested that there may be a certain correlation between final broth pH, lactonic SL and Lac SL/tot SL ratio in RSHH. The software of Minitab 17 was used to fit the correlation and the fitting curves and equations were displayed as followed (Fig. 2).

As shown in Fig. 2, the test results of different lactonic SL and ratio of Lac SL/tot SL are in good correlation with the variations of final broth pH values (p = 0.000 < 0.005). Both lactonic SL and Lac SL/tot SL ratio was negatively correlated to the final broth pH, with the linear fitting equation of y = 50.93 − 8.032x and y = 112.5 − 17.14x in the present study, separately. Although the mechanism and explicit relationship remains to be investigated, it did provide a good approach and idea to improve the output of different SLs. On the basis of above experiment results, choosing certain pH regulator to adjust broth pH and achieve large amount of lactonic SL, acidic SL or SLs with appropriate ratio would be possible. However, the complex regulation mechanism of broth pH on SL accumulation at different levels needs further explore and study.

## Discussion

Generally, glucose is common used as hydrophilic carbon source for SL production. Considering glucose is rather rarely found in larger quantities in wastes, which means non-recyclable and a high costs in SL production. In the present comparison, SL yield in non-glucose medium with different hydrophobic carbon sources, nitrogen sources and culture methods by various microbes are compared and exhibited in Table 6.

**Table 6:** Microorganisms, culture conditions, and SL production in non-glucose medium reported in previous references

Microorganism	Hydrophilic C source (g/L)	Hydrophobic C source (g/L)	N source (g/L)	Culture	T (°C)/t (h)	Yield (g/L)	Country	Reference
*T. bombicola* ATCC 22214 (Now known as *C. bombicola* ATCC 22214)	Fructose (20)	–	YE (10.0)	CF	30/72	4.10	Germany	Göbbert et al. (1984)
	Mannose (20)					4.90		
	Saccharose (20)					3.20		
	Maltose (20)					2.00		
	Raffinose (20)					4.10		
*C. bombicola* ATCC 22214	Sucrose (100)	Sunflower oil	YE (2.5)	CF	30/144	33.00	Czechoslovakia	Klekner et al. (1991)
			YE (5.0)			9.00		
			YE (10.0)			13.00		
			YE (20.0)			17.00		
*T. bombicola* ATCC 22214	Lactose (100)	Olive oil	YE (2.5–3.0)	b	30/192	46.46	Canada	Zhou and Kosaric (1993)
	Galactose (100)	Olive oil				24.41		
	Sucrose (100)	Safflower oil				58.32		
*C. bombicola* ATCC 22214	Lactose (100)	Canola oil	YE (4.0)	CF	30/192	90–110	Canada	Zhou and Kosaric (1995)
*C. bombicola* ATCC 22214	Deproteinized whey concentrate [lactose (100)]	Rapeseed oil	YE (4.0)	F-b	26/168	280.00	Germany	Daniel et al. (1998a)
*C. bombicola* ATCC 22214	Deproteinized cheese whey concentrate [lactose (110)]	SCO & rapeseed oil	–	F-b	30/410	422.00	Germany	Daniel et al. (1998b)
*C. bombicola* ATCC 22214	Soy molasses [333 + 667, total sugar (300)]	Oleic acid	YE (2.5)	F-b	26/168	21.00	USA	Solaiman et al. (2004)
*C. bombicola* ATCC 22214	Honey (100)	Turkish corn oil	YE (10.0)	F-b	25/436	>400	Turkey	Pekin et al. (2005)
*C. bombicola* ATCC 22214	Biodiesel co-product stream (100 + 100)	–	YE (10.0)	b	26/168	60.00	USA	Ashby et al. (2005)
*C. bombicola* ATCC 22214	Glycerol (100)	Methyl-soyate	YE (10.0)	F-b	27/168	46.00	USA	Ashby et al. (2006)
		ethyl-soyate				42.00		
		propyl-soyate				18.00		
*C. bombicola* ATCC 22214	Soy molasses [333 + 667, total sugar (300)]	Oleic acid	YE (10.0)	F-b	26/168	75.00	USA	Solaiman et al. (2007)
*C. bombicola* ATCC 22214	Sugarcane molasses (100)	Soybean oil	–	b	30/120	23.25	India	Daverey and Pakshirajan (2009)
*S. bombicola* NRRL Y-17069	Deproteinized whey (90) & glu (10)	Oleic acid	YE (2.0)	b	30/192	23.29	India	Daverey and Pakshirajan (2010)
				CF		25.54		
*C. bombicola* ATCC 22214	Sugarcane molasses (50)	Synthetic dairy wastewater & soybean oil	–	b	30/192	38.76	India	Daverey et al. (2011)
*S. bombicola* NBRC 10243	Sugarcane molasses [total sugar (150)]	–	–	F-b	25/120	14.40	Japan	Takahashi et al. (2011)
*S. bombicola* ATCC 22214	Sweetwater [glycerol (150)]	Sunflower oil	YE (4.0)	CF	30/200	6.60	India	Wadekar et al. (2012)
*W. domercqiae* var. *sophorolipid* CGMCC 1576	Delignined corncob residue hydrolysate [glu (60)]	Single cell oil	YE (3.0)	CF	30/168	42.06	China	Ma et al. (2013)
*C. bombicola* ATCC 22214	Sweet sorghum bagasse hydrolysate [glu (100)]	Soybean oil	YE (10.0)	CF	25/240	84.60	USA	Samad et al. (2014)
	corn fiber hydrolysate [glu (100)]					15.60		
*S. bombicola* ATCC 22214	Glycerol 150	Castor oil	–	CF	30/200	2.70	India	Bhangale et al. (2014)
*S. bombicola* NBRC 10243	Corncob hydrolysate [glu (45)]	Olive oil	YE (1.0)	CF	25/168	43.80	Japan	Konishi et al. (2015)
*W. domercqiae* var. *sophorolipid* CGMCC 1576	Rice straw holocellulose hydrolysate [glu (60)]	Oleic acid	YE (3.0)	CF	30/168	53.70	China	Present study

*T* temperature, *t* time, *F*-*b* fed-batch, *b* batch, *CF* continuous fermentor, *YE* yeast extract, *glu* glucose

As indicated in Table 6, yeasts, especially *Candida bombicola* ATCC 22214, are the most common strains for SLs production in various fermentation cultures. The production is significantly lower when only one carbon source was supplied than two types of carbon source were provided (Göbbert et al. 1984). The highest SL production obtained was 422 g/L by using deproteinized cheese whey concentrate and SCO & rapeseed oil as combined carbon sources, achieved by *C. bombicola*, with the fermentation time of 410 h and culture mode of fed-batch (Daniel et al. 1998b).

Several of cheap substrates can act as hydrophilic carbon sources, such as cheese whey, soy molasses, honey, glycerol, sugarcane molasses, sweet water, DCCR, CCR and RSH, etc. The main aims of all the attempts are to reduce substrate costs and increase SL yields. However, lower yields always were observed when culture mode of batch was used. Fed-batch (F-b) and continuous fermentation (CF) are beneficial for SL accumulation than batch. Yeast extract with different contents are common used nitrogen source in the listed references. Currently, lignocellulosic materials are potential substrates for SL production and relative high yields could be obtained. Further optimization on the pretreatment of lignocellulose, culture modes and reactors would improve the fermentation results even more.

Series of reports showed that blockage of the lignin droplets on the surface of the cellulose was the main cause of cellulase inhibition. Besides, the intensive structure of rice straw also hinders the attack of cellulase (Xing et al. 2012; Li et al. 2014). Nevertheless, some small molecule inhibitors would be generated during the pretreatment process and further detoxification treatment would be needed to remove the inhibiting effects on cell growth and product accumulation. However, for pretreatment of SO_3_ micro-thermal explosion on rice straw, our previous study indicated that the lignin content was dropped dramatically and there are no or little fermentation inhibitors in RSHH (Yao and Li 2013; Wang et al. 2015). And the pretreated rice straw had been directly applied for lipids (Yao et al. 2012), ethanol (Yao and Li 2013) etc. production. These findings are relative significant as it could simplify the lignocellulose utilization process since the detoxification treatment is not required.

Various factors were reported to affect sophorolipid accumulation, including C/N ratio, nitrogen source addition, temperature and oxygen supply (Stüwer et al. 1987). Poor research was related to the correlation between final broth pH and sophorolipid production or lac SL/tot SL ratio. In view of this, the effects of inoculum dose, nitrogen source (yeast extract) and pH regulator additions on sophorolipid exting was studied systematically.

As the increase of inoculum dose, the biomass was increased and the final broth pH was decreased. This phenomenon was usually ascribed to the increasing organic acid amount generated during more prosperous biological metabolism (Gupta and Prabhune 2012). Interestingly, lactonic SL and lac SL/tot SL ratio were negatively related to the final broth pH. It might be due to the induced effects of broth pH on lactonesterase activity or esterification process occurred in fermentation medium, and the specific mechanism was still remained explored.

Nitrogen type and content were reported to have extraordinary influences on SLs production and composition (Cooper and Paddock 1984; Zhou et al. 1992; Casas and García-Ochoa 1999; Ma et al. 2011). Generally, SLs production was initiated at the time of nitrogen source was exhausted and production of lactonic SL strongly depended on nitrogen source (Göbbert et al. 1984). The maximum yield, 53.70 g/L total SL and 22.32 g/L lactonic SL was obtained with the YE addition of 0.15 and 0.05 %, respectively. It indicates that SL production and ratio of lac SL/tot SL can be changed by different YE levels for suitable use. Except for the one without YE supplement (YE 0.00 %), the final broth pH was increased with the YE addition increasing, within the scope of 4.03–4.77. The results demonstrated that YE of appropriate contents, reached to appropriate final broth pH, would be more suitable for total SL or lactonic SL production.

Opposite to *C. apicola* (Stüwer et al. 1987) and *W. domercqia* (Ma et al. 2011), adjustment of pH by NaOH or Na_3_C_6_H_5_O_7_·2H_2_O showed a decrease in lactonic SL production by *W. domercqia* in RSHH. Furthermore, a strong negative correlation existing between final broth pH and lactonic SL or Lac SL/tot SL ratio was found. It demonstrated the feasibility of SLs obtainment with proper Lac SL/tot SL ratio for specific purpose and scope in a simple, cost efficient and sustainable way.

Based on the above, inoculum dose, yeast extract addition and pH regulator can be regarded as factors involved in the complex regulation of sophorolipid accumulation, and the adjustment of final broth pH was the ultimate way to achieve suitable sophorolipid mixture. These factors affected the final broth pH may be closely related to biological metabolism of *W. domercqia*, biosynthesis of SL production and the enzymes activities in different conditions.

Exploring the cheaper substrate to instead of glucose and optimizing the fermentation process was the main aim of this work, of course, which was the same meaning and purpose to the similar articles. For the overview of SL yield in non-glucose medium with different hydrophobic carbon sources, nitrogen source additions and culture methods, a lot of cheap wastes were studied containing sugars, sugar-producing biomass and lignocellulose wastes from crops. Considering the Sustainable development, harmfulness on environment and economic benefits etc. factors, DCCR, CCR and RSH etc. lignocellulosic materials from crops were regarded the most potential substrates. Besides, we believe that the recycling of crop waste was the inevitable trend in the future.

## Abbreviations

SL: sophorolipid
Lac SL: lactonic sophorolipid
Acid SL: acidic sophorolipid
RSHH: rice straw holocellulose hydrolysate
DCCR: delignified corncob residue hydrolysate
CCR: corncob residue hydrolysate
SEM: scanning electron microscope
Glu: glucose
